# Development and validation of a prediction model for in-hospital mortality of patients with severe thrombocytopenia

**DOI:** 10.1038/s41598-022-10438-y

**Published:** 2022-04-15

**Authors:** Yan Lu, Qiaohong Zhang, Jinwen Jiang

**Affiliations:** Clinical Laboratory, DongYang People’s Hospital, 60 West Wuning Road, Dongyang, 322100 Zhejiang China

**Keywords:** Diseases, Haematological diseases

## Abstract

Risk stratification and prognosis evaluation of severe thrombocytopenia are essential for clinical treatment and management. Currently, there is currently no reliable predictive model to identify patients at high risk of severe thrombocytopenia. This study aimed to develop and validate a prognostic nomogram model to predict in-hospital mortality in patients with severe thrombocytopenia in the intensive care unit. Patients diagnosed with severe thrombocytopenia (N = 1561) in the Medical Information Mart for Intensive Care IV database were randomly divided into training (70%) and validation (30%) cohorts. In the training cohort, univariate and multivariate logistic regression analyses with positive stepwise selection were performed to screen the candidate variables, and variables with *p* < 0.05 were included in the nomogram model. The nomogram model was compared with traditional severity assessment tools and included the following 13 variables: age, cerebrovascular disease, malignant cancer, oxygen saturation, heart rate, mean arterial pressure, respiration rate, mechanical ventilation, vasopressor, continuous renal replacement therapy, prothrombin time, partial thromboplastin time, and blood urea nitrogen. The nomogram was well-calibrated. According to the area under the receiver operating characteristics, reclassification improvement, and integrated discrimination improvement, the nomogram model performed better than the traditional sequential organ failure assessment (SOFA) score and simplified acute physiology score II (SAPS II). Additionally, according to decision curve analysis, a threshold probability between 0.1 and 0.75 indicated that our constructed nomogram model showed more net benefits than the SOFA score and SAPS II. The nomogram model we established showed superior predictive performance and can assist in the quantitative assessment of the prognostic risk in patients with severe thrombocytopenia.

## Introduction

Platelets are produced by megakaryocytes in the bone marrow and participate in hemostasis and thrombosis. In healthy individuals, platelets exhibit normal physiological functions, and their number remains above 150 × 10^9^/L^1^. However, increased platelet consumption, impaired production, or severe destruction may lead to varying degrees of platelet reduction^2^. To date, there is no consistent definition of thrombocytopenia. Generally, thrombocytopenia is classified into mild (100–149 × 10^9^/L), moderate (50–99 × 10^9^/L) and severe (< 50 × 10^9^/L), according to the absolute number of platelets^3^.

Thrombocytopenia is common in critically ill patients. Studies have found that more than 60% of intensive care unit (ICU) patients develop thrombocytopenia^4^, with severe thrombocytopenia occurring in 2.3–27% of them^5^ As the most common cause of hemostatic disorders in ICUs, thrombocytopenia is associated with a higher risk of bleeding and blood transfusion^6^. Although the mechanisms leading to thrombocytopenia vary, severe thrombocytopenia indicates a worse clinical prognosis in critically ill patients^7,8^.

Identifying risk factors for mortality in patients with severe thrombocytopenia can help clinicians develop individualized treatment strategies and enhanced care management to reduce the risk of adverse events and improve patient outcomes. Thus, risk stratification and prognosis evaluation of patients with severe thrombocytopenia are essential for clinical treatment and management. Currently, there is currently no reliable predictive model that can be used to identify patients at a high risk of severe thrombocytopenia.

The visual nomogram is an intuitive and easy-to-use predictive tool whose performance has been validated in predictive models for multiple diseases^9,10^. This study aimed to develop and verify a nomogram model to predict mortality in patients with severe thrombocytopenia during ICU admission based on a large dataset of diagnosis and treatment from the Medical Information Mart for Intensive Care IV (MIMIC-IV) database.

## Results

### Patient characteristics

In total, 1,561 patients with severe thrombocytopenia were enrolled in this study and randomly divided into a training cohort of 1,093 patients and a validation cohort of 468 patients. The in-hospital mortality rate of the training cohort was 31.5%, and that of the validation cohort was 35.5%. As shown in Table 1, no significant differences were observed in any of the variables between the training and validation cohorts.

**Table 1:** Baseline characteristics of patients included in this study (N = 1561).

Variables	Training Cohort		Validation Cohort		*p*-value
	Survivors (n = 749)	Non-survivors (n = 344)	Survivors (n = 302)	Non-survivors (n = 166)	
**Age, years**	60 (50–69)	64.5 (55–75)	59 (51–68)	65 (55–74)	0.754
**Sex, n (%)**					0.253
Male	440 (58.7)	194 (56.4)	177 (58.6)	57 (34.3)	
Female	309 (41.3)	150 (43.6)	125 (41.4)	109 (65.7)	
**Weight, kg**	79.8 (67.4–94.3)	77.4 (64.8–94.6)	78.4 (65.7–91.4)	80.3 (69–96.3)	0.806
**Comorbidities, n (%)**					
Myocardial infarction	62 (8.3)	51 (14.8)	27 (8.9)	26 (15.7)	0.562
Cerebrovascular disease	62 (8.3)	47 (13.7)	26 (8.6)	19 (11.4)	0.828
Chronic pulmonary disease	163 (21.8)	79 (23.0)	55 (18.2)	33 (19.9)	0.139
Rheumatic disease	21 (2.8)	15 (4.4)	7 (2.3)	6 (3.6)	0.592
Liver disease	349 (46.6)	156 (45.3)	159 (52.6)	79 (47.6)	0.092
Diabetes	173 (23.1)	95 (27.6)	72 (23.8)	46 (27.7)	0.771
Renal disease	139 (18.6)	87 (25.3)	51 (16.9)	46 (27.7)	0.982
Malignant cancer	238 (31.8)	156 (45.3)	91 (30.1)	75 (45.2)	0.827
Sepsis	145 (19.4)	106 (30.8)	48 (15.9)	53 (31.9)	0.549
**Vital signs**					
Temperature, °C	36.9 (36.6–37.2)	36.7 (36.4–37.1)	36.9 (36.6–37.2)	36.7 (36.4–37.2)	0.380
Oxygen saturation, %	97.2 (95.7–98.5)	96.4 (94.6–98.1)	97.3 (95.9–98.6)	96.6 (94.6–98.1)	0.511
Heart rate, bpm	89.0 (78.1–100.3)	95.8 (82.7–108.5)	90.1 (77.0–101.6)	98.6 (84.1–110.6)	0.162
Mean arterial pressure, mmHg	76.0 (69.8–82.9)	71.0 (66.1–77)	75.9 (69.6–84.4)	74.1 (68.6–79.6)	0.103
Respiration rate, bpm	18.6 (16.4–21.7)	21.2 (18.1–25.1)	18.9 (15.9–22.7)	21.4 (18–25.3)	0.403
**Severity scores**					
SOFA	8 (6–11)	13 (10–16)	8 (6–11)	13 (9–16)	0.887
SAPS II	39 (30–49)	55 (45–67)	38.5 (29–49)	55 (46–66)	0.889
**Medical treatment, n (%)**					
Mechanical ventilation	133 (17.8)	122 (35.5)	51 (16.9)	59 (35.5)	0.941
Platelet transfusion	396 (52.9)	222 (64.5)	137 (45.4)	108 (65.1)	0.127
Vasopressor	217 (29.0)	247 (71.8)	95 (31.5)	99 (59.6)	0.714
Continuous renal replacement therapy	52 (6.9)	80 (23.3)	23 (7.6)	47 (28.3)	0.120
**Laboratory test results**					
White blood cell count, 10^9^/L	6.4 (3.2–11.1)	7.7 (3.1–14.3)	6.3 (3.2–11)	7.2 (2.8–13.6)	0.690
Hemoglobin, g/dL	9 (7.6–10.6)	8.8 (7.4–10.5)	9.1 (7.6–10.7)	8.4 (7.4–10.1)	0.914
Glucose, mg/dL	121 (102–164)	133 (101–177)	123 (104–165)	129 (97–182)	0.861
Creatinine, mg/dL	1 (0.7–1.6)	1.5 (1–2.6)	1.0 (0.7–1.6)	1.6 (1.0–2.7)	0.620
Sodium, mmol/l	138 (135–141)	138 (134–142)	138 (135–141)	138 (134–142)	0.619
Potassium, mmol/l	4.0 (3.6–4.5)	4.1 (3.6–4.7)	4 (3.6–4.4)	4.3 (3.7–5)	0.362
Prothrombin time, s	16 (13.6–19.1)	19.3 (15.6–25)	16.2 (13.7–20.3)	18.1 (15.2–23.7)	0.822
Partial thromboplastin time, s	33.6 (28.8–41.9)	38.5 (30.4–52.8)	33.8 (29.8–40.4)	39.5 (31.8–55.9)	0.374
International normalized ratio	1.5 (1.2–1.8)	1.7 (1.4–2.4)	1.5 (1.3–1.9)	1.8 (1.4–2.4)	0.325
Blood urea nitrogen, mg/dL	22 (13–38)	35.5 (22–58.5)	23 (13–38)	37 (24–56)	0.241
Bicarbonate, mmol/L	22 (19–24)	19 (15.5–23)	22 (18–25)	19 (17–23)	0.813
Chloride, mmol/L	105 (100–109)	103 (98–108)	104 (100–108)	105 (100–109)	0.463
Red blood cell distribution width, %	16.1 (14.6–18.3)	17.0 (15.2–19.4)	16 (14.8–17.8)	17.3 (15.2–20.1)	0.345
Alanine aminotransferase, IU/L	40 (21–95)	38.5 (21–87)	37 (21–94)	39 (24–85)	0.865
ICU stay, days	2.9 (1.8–5.5)	3.3 (1.8–7.2)	2.7 (1.8–5.0)	3.7 (2.1–8.1)	0.565

SOFA: Sequential Organ Failure Assessment; SAPS II: Simplified Acute Physiology Score II; ICU: intensive care units.

### Screening of prognostic factors

As shown in Table 2, in the univariate logistic regression analysis, 23 variables were significantly related to the in-hospital mortality rate in severe thrombocytopenia (*p* < 0.05). Based on the multivariate logistic regression analysis with positive stepwise selection, the following were identified as independent factors for in-hospital mortality in patients with severe thrombocytopenia: age, cerebrovascular disease, malignant cancer, oxygen saturation, heart rate, respiration rate, mean arterial pressure, mechanical ventilation, vasopressors, continuous renal replacement therapy (CRRT), prothrombin time (PT), partial thromboplastin time (PTT), and blood urea nitrogen (BUN). Moreover, the variance inflation factor (VIF) of all variables was < 10, indicating no significant collinearity.

**Table 2:** Univariate and multivariate logistic regression analysis in the training cohort (n = 1093).

Variables	Univariate model			Multivariable model		
	Odds ratio	95% confidence interval	*p*-value	Odds ratio	95% confidence interval	*p*-value
Age	1.022	1.013–1.031	< 0.001	1.022	1.010–1.034	< 0.001
Sex (Male vs. Female)	0.908	0.702–1.176	0.465	–	–	–
Weight, Kg	0.999	0.993–1.005	0.728	–	–	–
Myocardial infarction (Yes vs. No)	1.929	1.299–2.863	0.001	–	–	–
Cerebrovascular disease (Yes vs. No)	1.754	1.172–2.623	0.006	2.548	1.544–4.203	< 0.001
Chronic pulmonary disease (Yes vs. No)	1.072	0.790–1.454	0.657	–	–	–
Rheumatic disease (Yes vs. No)	1.581	0.805–3.105	0.209	–	–	–
Liver disease (Yes vs. No)	0.951	0.736–1.229	0.701	–	–	–
Diabetes (Yes vs. No)	1.270	0.949–1.700	0.107	–	–	–
Renal disease (Yes vs. No)	1.486	1.095–2.015	0.011	–	–	–
Malignant cancer (Yes vs. No)	1.782	1.371–2.316	< 0.001	2.329	1.627–3.335	< 0.001
Sepsis (Yes vs. No)	1.855	1.386–2.484	< 0.001	–	–	–
Temperature, °C	0.958	0.903–1.016	0.152	–	–	–
Oxygen saturation, %	0.842	0.797–0.890	< 0.001	0.893	0.835–0.955	0.001
Heart rate, bpm	1.026	1.018–1.034	< 0.001	1.022	1.011–1.033	< 0.001
Mean arterial pressure, mmHg	0.953	0.940–0.966	< 0.001	0.972	0.956–0.989	0.001
Respiration rate, bpm	1.116	1.085–1.148	< 0.001	1.057	1.019–1.095	0.003
Mechanical ventilation (Yes vs. No)	2.545	1.905–3.400	< 0.001	1.718	1.179–2.504	0.005
Platelet transfusion (Yes vs. No)	1.622	1.246–2.111	< 0.001	–	–	–
Vasopressor (Yes vs. No)	6.243	4.704–8.285	< 0.001	3.819	2.677–5.448	< 0.001
Continuous renal replacement therapy (Yes vs. No)	4.062	2.787–5.920	< 0.001	1.626	1.012–2.614	0.045
White blood cell count, 10^9^/L	0.998	0.993–1.004	0.564	–	–	–
Hemoglobin, g/dL	1.000	0.944–1.059	0.994	–	–	–
Glucose, mg/dL	1.001	0.999–1.003	0.196	–	–	–
Creatinine, mg/dL	1.231	1.137–1.333	< 0.001	–	–	–
Sodium, mmol/l	0.989	0.968–1.010	0.313	–	–	–
Potassium, mmol/l	1.200	1.039–1.385	0.013	–	–	–
Prothrombin time, s	1.082	1.061–1.103	< 0.001	1.065	1.040–1.090	< 0.001
Partial thromboplastin time, s	1.010	1.005–1.014	< 0.001	1.008	1.002–1.014	0.007
International normalized ratio	1.489	1.281–1.730	< 0.001	–	–	–
Blood urea nitrogen, mg/dL	1.019	1.014–1.024	< 0.001	1.016	1.010–1.021	< 0.001
Bicarbonate, mmol/L	0.919	0.895–0.943	< 0.001	–	–	–
Chloride, mmol/L	0.978	0.961–0.995	0.021	–	–	–
Red blood cell distribution width, %	1.084	1.041–1.128	< 0.001	–	–	–
Alanine aminotransferase, IU/L	1.000	1.000–1.000	0.462	–	–	–

### Construction and verification of nomogram

**Figure 1:**
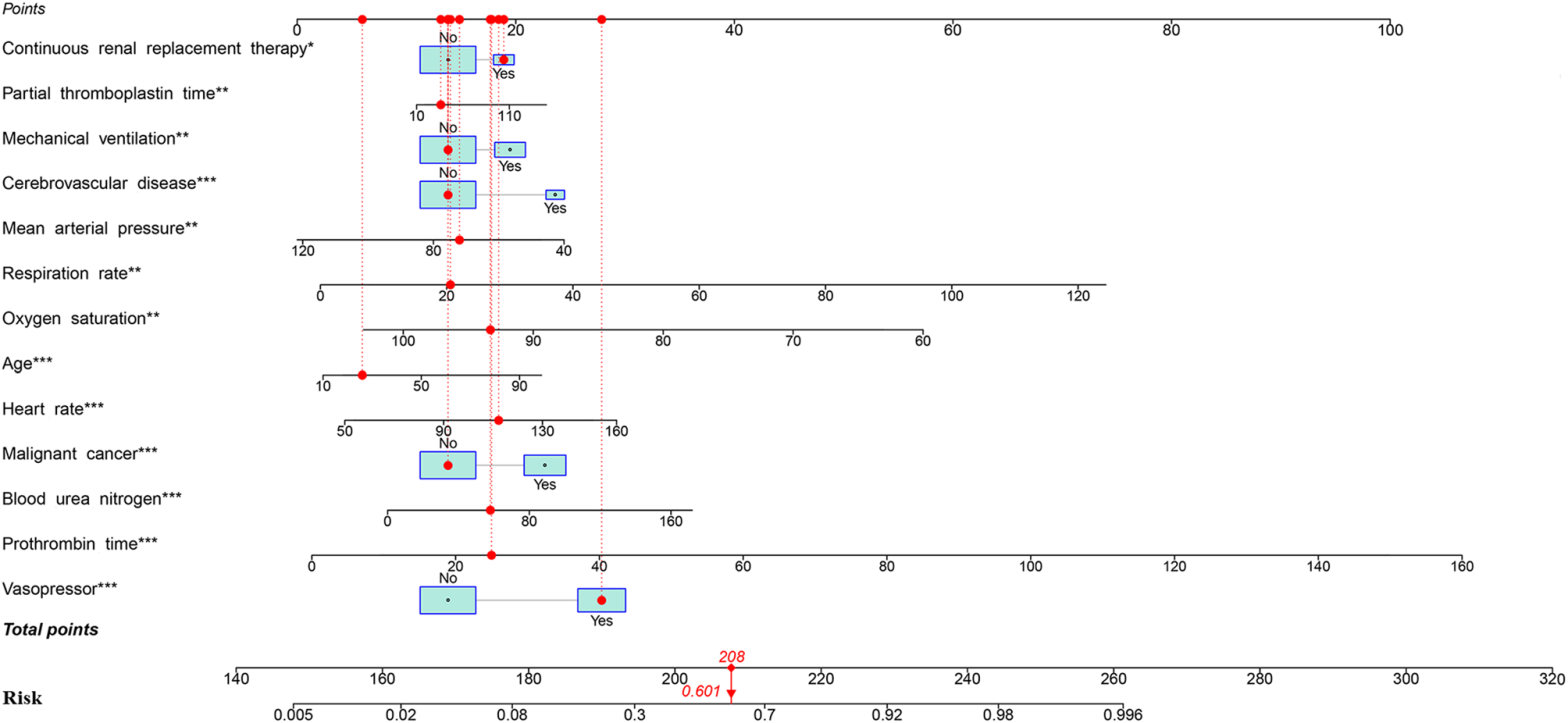
Established nomogram for predicting the risk of in-hospital mortality in patients with severe thrombocytopenia. Each variable value corresponds to the “point” axis to determine the corresponding score, and the total points were obtained by adding the scores of all variables. To determine the risk of in-hospital mortality, locate the total score on the "total score" axis, and follow vertically downwards to find the risk of in-hospital mortality on the "risk" axis. *p < 0.05, * *p < 0.01 and ***p < 0.001.

Based on the results of the multivariate logistic regression analysis, we constructed a nomogram to predict the in-hospital mortality of patients with severe thrombocytopenia (Fig. 1). For patients aged 26 years who were prescribed vasopressor agents, underwent CRRT, and presented oxygen saturation of 93.3%; mean arterial pressure of 72 mmHg; heart rate of 112 bpm; respiration rate of 21 bpm; PT of 25 s; PTT of 36 s; and BUN of 58 mg/dL, the total number of points on the nomogram was 208, which predicted an in-hospital mortality of 0.601.

**Figure 2:**
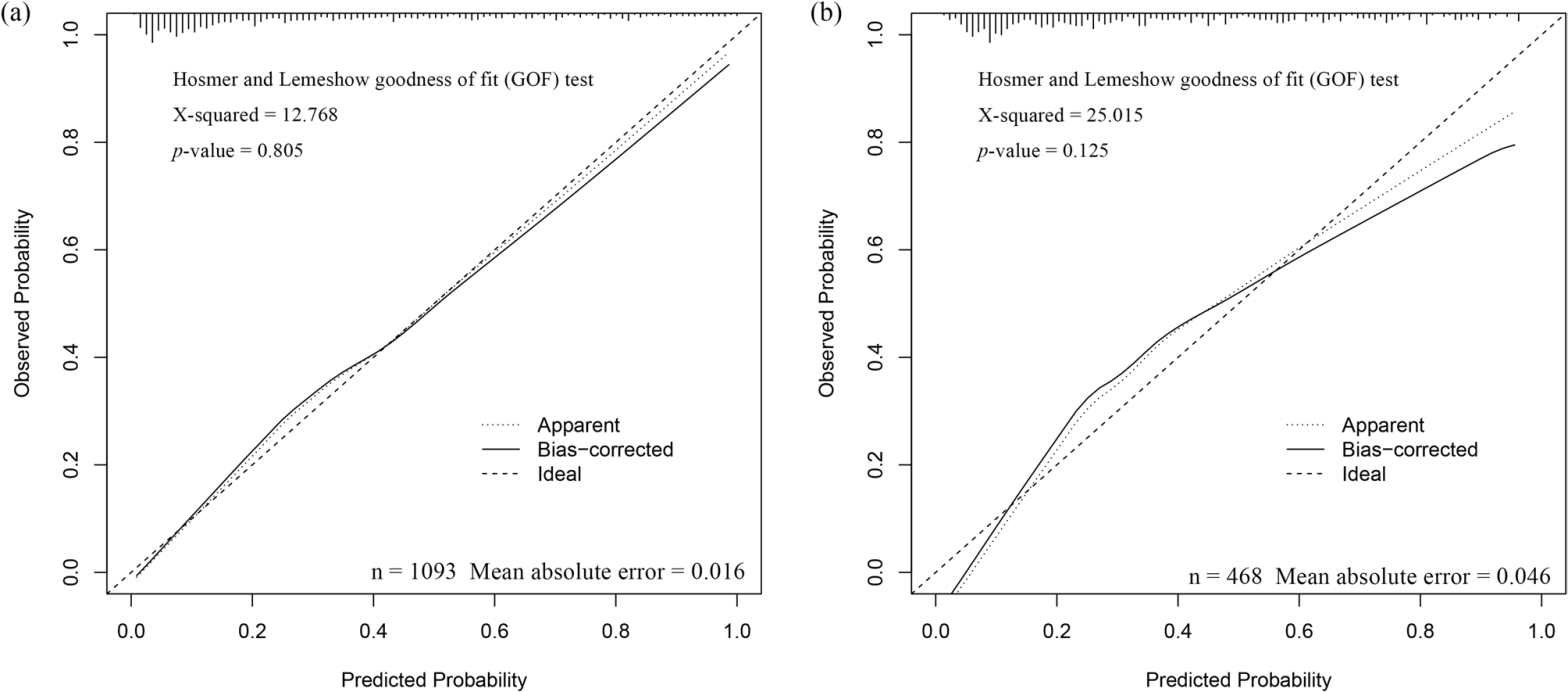
Calibration curve of the established nomogram. (**a**) Training cohort, (**b**) verification cohort.

The C-indices of the nomogram in the training and validation cohorts were 0.846 (95% confidence interval [CI]: 0.806–0.886) and 0.828 (95% CI: 0.791–0.865), respectively, indicating excellent prediction performance. In addition, the Hosmer–Lemeshow goodness-of-fit test indicated that the predicted outcome of the patient agreed with the actual outcome (Fig. 2).

**Figure 3:**
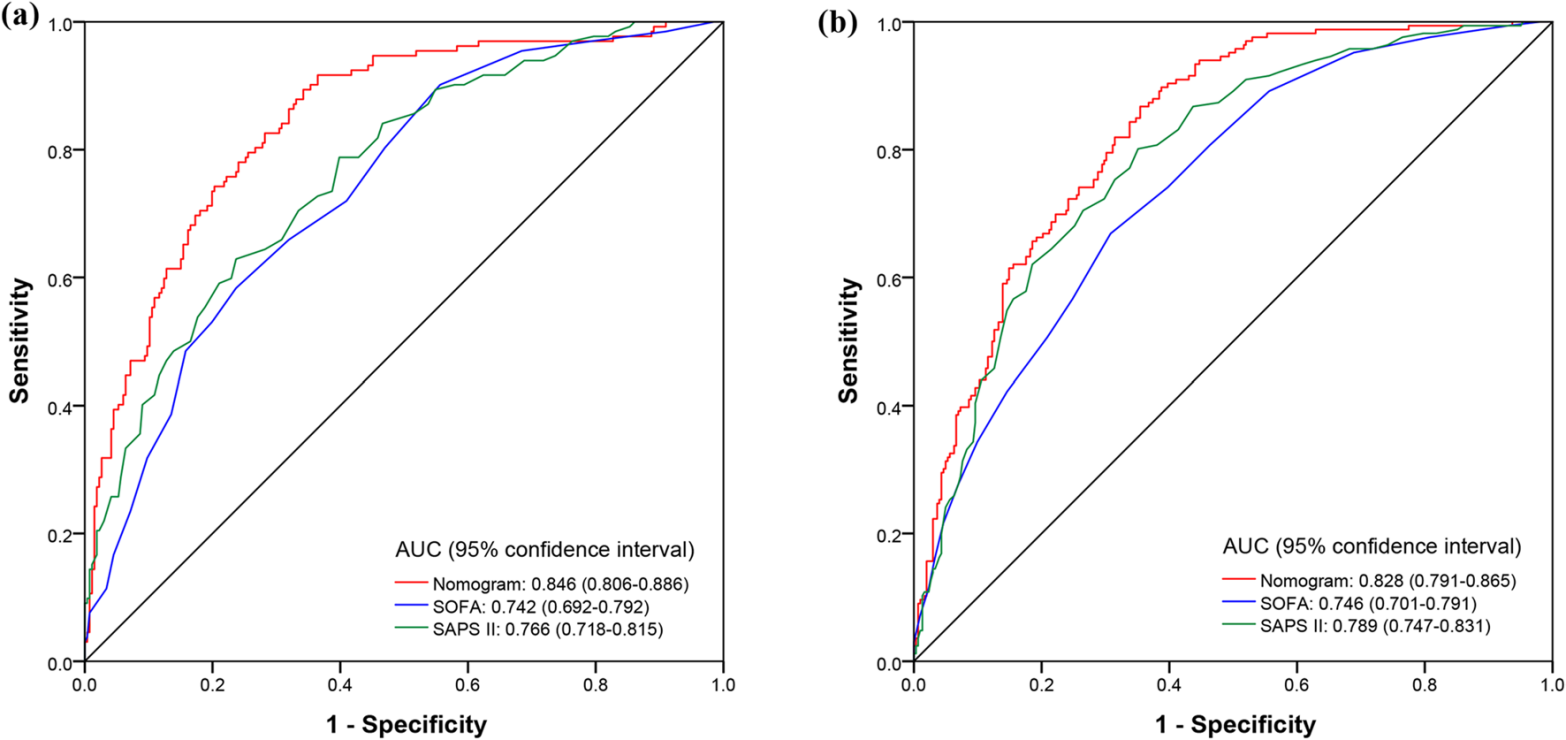
ROC curve of the established nomogram, SOFA, and SAPS II. (**a**) Training cohort, (**b**) verification cohort.

To compare the predictive performance of the nomogram model and the most commonly used severity assessment scales in the ICU, we constructed receiver operating characteristics (ROC) curves (Fig. 3). The areas under the ROC curve of the nomogram in the training and validation queues were higher than those of sequential organ failure assessment (SOFA) score and simplified acute physiology score II (SAPS II). In addition, integrated discrimination improvement (IDI) and net reclassification improvement (NRI) reflected the intuitive improvement in the nomogram. Compared with those of the SOFA score, the NRI and IDI values of the nomogram in the training cohort were 0.272 (95% CI = 0.190–0.355) and 0.177 (95% CI = 0.148–0.206), respectively, whereas those in the validation cohort were 0.250 (95% CI = 0.129–0.371) and 0.129 (95% CI = 0.087–0.171), respectively. Compared with SAPS II, the NRI and IDI values of the nomogram in the training cohort were 0.282 (95% CI = 0.201–0.362) and 0.137 (95% CI = 0.107–0.166), respectively; in the validation cohort, they were 0.168 (95% CI = 0.061–0.276) and 0.078 (95% CI = 0.039–0.117), respectively.

### Clinical application of the nomogram

**Figure 4:**
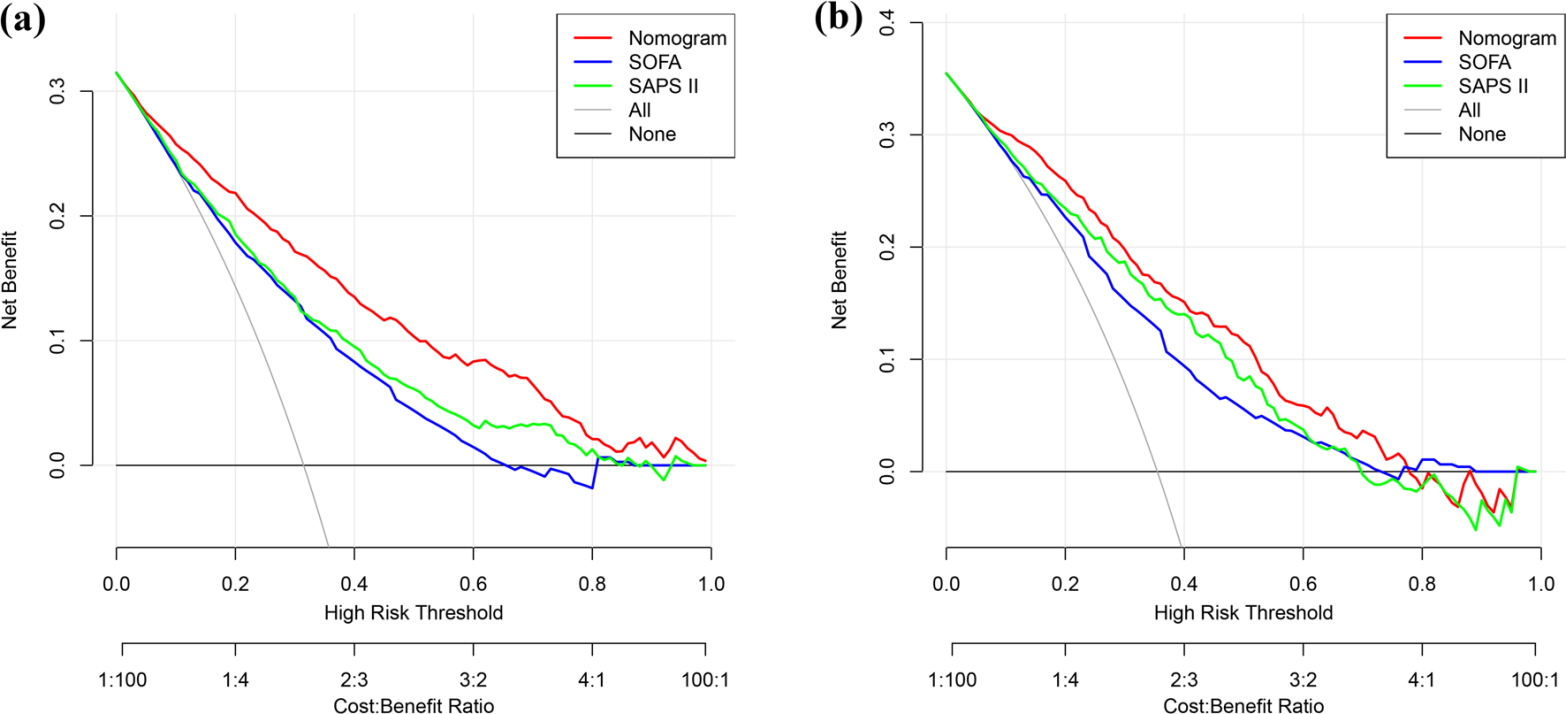
Decision curve analysis of the established nomogram, SOFA, and SAPS II. (**a**) Training cohort, (**b**) verification cohort.

As shown in Fig. 4, regardless of the cohort (training or validation), a threshold probability between 0.1 and 0.75 indicated that our constructed nomogram model showed more net benefits than the SOFA score and SAPS II. For example, according to Fig. 4a, if the risk threshold is set to 0.4 when we individualize and intensify care for patients with a greater than 40% risk of in-hospital mortality, for every 100 patients who use the nomogram model, 14 gain benefits without incurring losses to others. In contrast, only eight and ten patients using the SOFA score and SAPS II achieved a net benefit, respectively.

## Discussion

Patients with thrombocytopenia are at an increased risk of bleeding, and even a mild or moderate decrease in platelet count in critically ill patients cannot be ignored^11^. Severe thrombocytopenia can no longer be regarded as a comorbidity of other diseases, as it severely affects patient management and restricts doctors from providing invasive intervention. Therefore, it was necessary to develop a specific model to predict patient prognosis.

To the best our knowledge, this is the first study to develop and validate a predictive model for in-hospital mortality in patients with severe thrombocytopenia. We revealed the following as independent risk factors for in-hospital mortality in patients with severe thrombocytopenia: age, presence of cerebrovascular disease, malignant cancer, oxygen saturation, heart rate, respiration rate, mean arterial pressure, mechanical ventilation, vasopressor use, CRRT; PT, PTT, and BUN. These risk factors were used to construct a nomogram model, which was verified and evaluated using ROC curve, calibration curve, IDI, NRI, and decision curve analysis. We believe that the nomogram model has good predictive performance and clinical application value.

In the prognostic models of different diseases, age, as a non-interventional factor, has been reported as an independent risk factor for most diseases^12,13^. Generally, the decline in the body's immunity with age is inevitable^14^. Additionally, the existence of non-interventional factors affects the net benefit of clinical intervention based on this nomogram.

Vital signs beyond the normal range indicate that the body is making compensatory adjustments. However, once out of control, the patients’ condition becomes serious, and clinical interventions, such as mechanical ventilation and vasopressor agents, are necessary. Comorbidities affect patients in many ways, and this may be due to the absence of platelet homeostasis. Cardiovascular diseases often require antiplatelet therapy, which contradicts the treatment of patients with severe thrombocytopenia^15^. The immune system of patients with malignant tumors is severely damaged, and thrombocytopenia is the main side effect of cancer treatment^16^.

From the nomogram model, it is clear that PT, PTT, and BUN are laboratory parameters worth paying attention to. PT and PTT reflect the function of the coagulation system. Patients with severe thrombocytopenia have microvascular failure, organ dysfunction, and blood coagulation disorders which increase the risk of bleeding^17^. BUN is an indicator of renal function; studies have found that it is an independent risk factor for the prognosis of critically ill patients, especially those with cardiogenic diseases^18,19^. Our study found that BUN is independently related to the prognosis of patients with severe thrombocytopenia, possibly because patients with renal failure also have complex hemostatic disorders^20,21^.

A nomogram is a common visual presentation for predictive models. Compared with the traditional critically ill patient scoring systems (SOFA and SAPS II), risk stratification based on our nomogram model showed a higher clinical benefit.

However, our findings had some limitations. First, because the data were extracted from the MIMIC-IV database, though the nomogram model passed verification in the validation cohort, its universality is limited, and verification using multi-center data is required. Second, because of the retrospective study design, potential selection bias is unavoidable, and the results should be interpreted with caution. Third, it is undeniable that there may be variables that were not included in the model, which have significant effects on in-hospital mortality in patients with severe thrombocytopenia. Fourth, the nomogram model comprises multiple variables and, in practice, it is not suitable for patients with missing variables. Therefore, we recommend deciding whether to use this model to assess patient outcomes either before or at the time of patient admission to the ICU, as opposed to waiting for more than 24 h following admission. Although the nomogram model could provide an important reference for clinical diagnosis and treatment, it could not accurately predict prognosis.

The nomogram model we developed can help quantitatively assess the prognostic risk factors in patients with severe thrombocytopenia. This model has superior predictive performance and can provide a good reference for evaluating the in-hospital mortality rate in patients with severe thrombocytopenia. However, multi-center prospective studies are required for further verification.

## Methods

### Data source

The MIMIC-IV database contains all data from intensive care patients admitted to the ICU or emergency department of Beth Israel Deaconess Medical Center (Boston, MA) from 2008 to 2019^22^. The collection of raw data, as well as the establishment of the database, was approved by the Institutional Review Board of Beth Israel Deaconess Medical Center and the Massachusetts Institute of Technology (Cambridge, MA). Because all medical data were deidentified, the requirement for individual patient consent was waived. In accordance with the data usage agreement, Lu passed the training for protecting human research participants (certificate number: 35953547) and was responsible for the acquisition and analysis of research data. All methods were performed in accordance with the relevant guidelines and regulations.

### Study population

Severe thrombocytopenia was defined as the lowest platelet value < 50 × 10^9^/L within 24 h of ICU admission. Data on 2434 patients with severe thrombocytopenia were extracted from all patients admitted to the ICU. Some data were excluded based on the following criteria: (1) If the same patient was admitted multiple times, only the information of the patient's first admission was retained; (2) Patients who were younger than 18 years old; (3) Patients who were hospitalized in the ICU for < 24 h; (4) Patients who received platelet transfusion before ICU admission; and (5) Patients with missing clinical data > 20%. (Supplementary Fig. 1).

### Data extraction

We used the structured query language in PostgreSQL 10 software to extract clinical data from the final study population. The following data were collected (results of multiple measurements were included as the initial value or the mean value as required): (1) demographics including age, sex, and weight measured for the first time after ICU admission; (2) comorbidities; (3) the mean value of vital sign measurements within 24 h of ICU admission; (4) severity scores on the first day of ICU admission; (5) medical treatment; and (6) the initial value of laboratory measurements obtained within 24 h of ICU admission. When the proportion of missing values for a variable was less than 20% (Supplementary Table 1), the missing part of the variable was filled in the training cohort and the validation cohort using multiple imputations, respectively. Otherwise, the variables were excluded. To reduce the influence of extreme values on the conclusions of the study, continuous variables were winsorized by 1% on both sides^23^.

### Statistical analyses

Patients with severe thrombocytopenia included in the study were randomly divided into training (70%) and validation (30%) cohorts. After analyzing the distribution of continuous variables using the Shapiro–Wilk test, the normally distributed variables are presented as the mean ± standard deviation. Non-normally distributed variables are represented by the median and interquartile range. Categorical variables are expressed as quantities (percentages). The differences between groups of continuous variables were compared using unpaired Student's *t*-test and Wilcox test. The chi-square test was used for categorical variables.

IBM Statistical Package for the Social Sciences for Windows (version 22.0. Armonk, NY: IBM Corp) and R (version 4.1.0) software were used for data analysis and drawing graphs. In the training cohort, univariate logistic regression analysis was initially used to screen for candidate variables. Then, according to the value minimization principle of Akaike information criteria^24^, multivariate logistic regression analysis with positive stepwise selection was performed on the statistically significant variables (*p* < 0.05). The VIF tested the collinearity between variables, whose acceptable range is < 10^25^. The "rms" package of R software was used to convert the results into nomograms.

To test the clinical application performance of the model, both the training and validation cohorts were drawn with ROC curves. The performance of the SOFA score and SAPS II were compared with the performance of the new model. We used the NRI and IDI to more objectively evaluate the improvement of the new model^26^. The calibration curve used the Hosmer–Lemeshow goodness-of-fit test to reflect the consistency between the actual and the predicted probabilities^27^. Decision curve analysis was performed to assess the net clinical benefits of the new model under different thresholds^28^.

The outcome variable was all-cause in-hospital mortality in patients with severe thrombocytopenia. This study was based on the transparent reporting of individual prognosis or diagnosis (TRIPOD) of multivariate predictive models guidelines for analysis and reporting^29^.

### Ethics approval and consent to participate

The collection of raw data, as well as the establishment of the database, was approved by the Institutional Review Board of Beth Israel Deaconess Medical Center and the Massachusetts Institute of Technology (Cambridge, MA). Since all medical data were deidentified, the requirement for individual patient consent was waived. In accordance with the data usage agreement, Lu passed the training for protecting human research participants.

## Supplementary Information

Supplementary Information.

## Data Availability

All available data were obtained from MIMIC-IV database. The original contributions presented in the study are included in the article/supplementary material; further inquiries can be directed to the corresponding author/s.

## Abbreviations

BUN: Blood urea nitrogen
CRRT: Continuous renal replacement therapy
ICU: Intensive care units
IDI: Integrated Discrimination Improvement
MIMIC-IV: Intensive Care Medical Information Market IV
NRI: Net reclassification improvement
PTT: Partial thromboplastin time
PT: Prothrombin time
ROC: Receiver operating characteristics
RDW: Red blood cell distribution width
SAPS II: Simplified acute physiology score II
SOFA: Sequential organ failure assessment
VIF: Variance inflation factor
