# Supplementary material for: Development and validation of a prediction model for in-hospital mortality of patients with severe thrombocytopenia
Source: Sci Rep. 2022 Apr 15;12:6316. doi: 10.1038/s41598-022-10438-y (PMC9012749; doi:10.1038/s41598-022-10438-y)
Supplement: Supplementary file 1 — Supplementary Information. [file 41598_2022_10438_MOESM1_ESM.pdf]

Development and validation of a prediction model for in-hospital mortality of patients with severe thrombocytopenia

Yan Lu[1*], Qiaohong Zhang[1], Jinwen Jiang[1]

[1] Clinical Laboratory, DongYang People's Hospital, 60 West Wuning Road, Dongyang 322100, Zhejiang, China

*** Corresponding Author:**
Yan Lu
luyan11219@hotmail.com

Supplementary Table 1 The missingness of raw data in this study.

Variables	Training Cohort （n=1093）		Validation Cohort （n=468）		Total （n=1561）	
	Number of missing	Missing proportion (%)	Number of missing	Missing proportion (%)	Number of missing	Missing proportion (%)
Age	0	0	0	0	0	0
Sex	0	0	0	0	0	0
Weight	3	0.27	2	0.43	5	0.32
Myocardial infarction	0	0	0	0	0	0
Cerebrovascular disease	0	0	0	0	0	0
Chronic pulmonary disease	0	0	0	0	0	0
Rheumatic disease	0	0	0	0	0	0
Liver disease	0	0	0	0	0	0
Diabetes	0	0	0	0	0	0
Renal disease	0	0	0	0	0	0
Malignant cancer	0	0	0	0	0	0
Sepsis	0	0	0	0	0	0
Temperature	53	4.85	19	4.06	72	4.61
Oxygen saturation	25	2.29	15	3.21	40	2.56
Heart rate	3	0.27	1	0.21	4	0.26
Mean arterial pressure	26	2.38	11	2.35	37	2.37

Respiration rate	3	0.27	2	0.43	5	0.32
SOFA	0	0	0	0	0	0
SAPS II	0	0	0	0	0	0
Mechanical ventilation	0	0	0	0	0	0
Platelet transfusion	0	0	0	0	0	0
Vasopressor	0	0	0	0	0	0
Continuous renal replacement therapy	0	0	0	0	0	0
White blood cell count	91	8.33	50	10.68	141	9.03
Hemoglobin	35	3.20	15	3.21	50	3.20
Glucose	27	2.47	12	2.56	39	2.50
Creatinine	32	2.93	12	2.56	44	2.82
Sodium	24	2.20	10	2.14	34	2.18
Potassium	25	2.29	20	4.27	45	2.88
Prothrombin time	66	6.04	34	7.26	100	6.41
Partial thromboplastin time	78	7.14	33	7.05	111	7.11
International normalized ratio	67	6.13	24	5.13	91	5.83
Blood urea nitrogen	29	2.65	12	2.56	41	2.63
Bicarbonate	24	2.20	13	2.78	37	2.37
Chloride	29	2.65	9	1.92	38	2.43
Red blood cell distribution width	26	2.38	10	2.14	36	2.31

Alanine aminotransferase	183	16.74	77	16.45	260	16.66
ICU stay	0	0	0	0	0	0

SOFA: Sequential Organ Failure Assessment; SAPS II: Simplified Acute Physiology Score II; ICU: intensive care units.

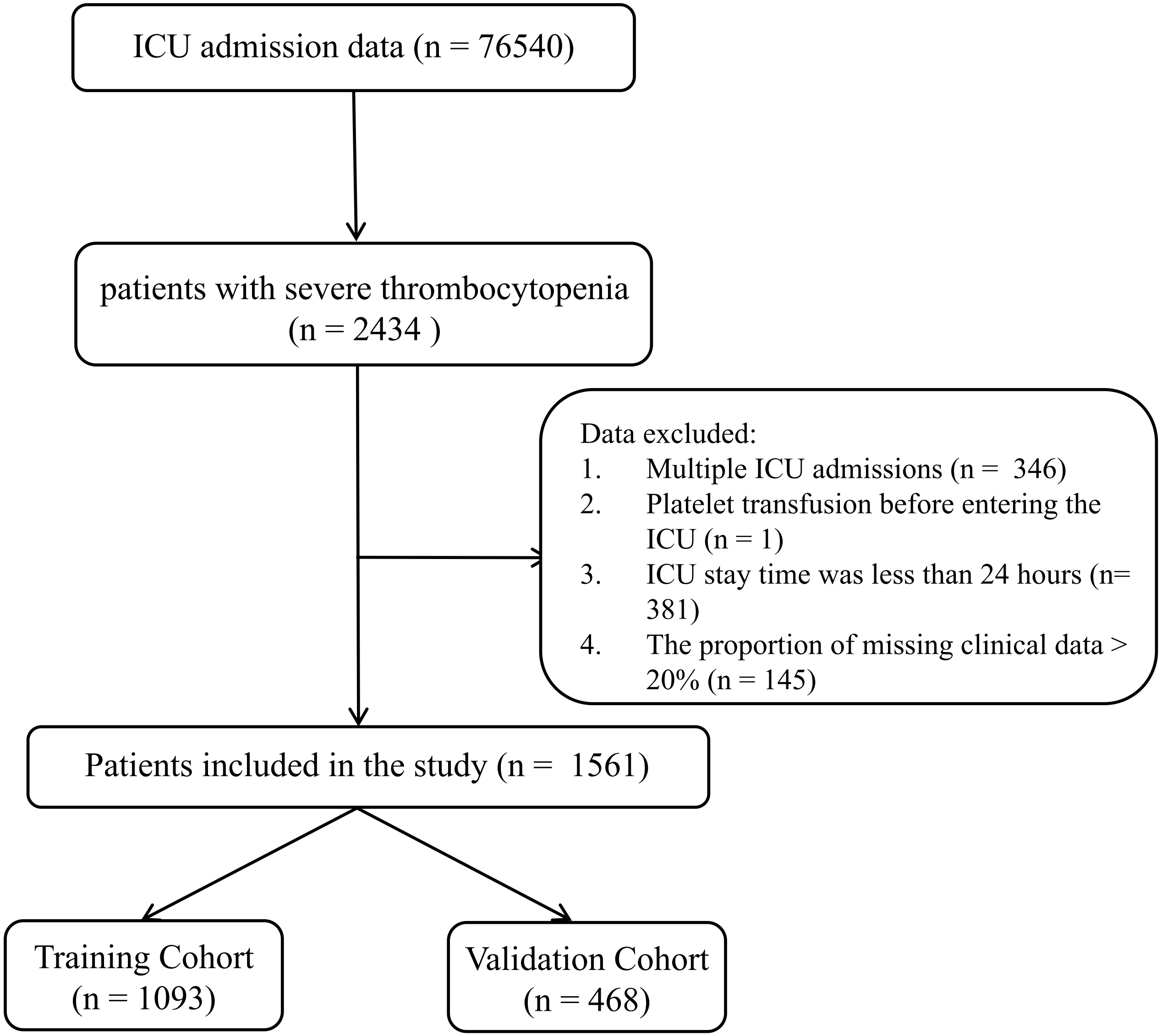

Supplementary Figure 1. Research data screening flowchart.
